# IL-8 and eNOS polymorphisms predict bevacizumab-based first line treatment outcomes in *RAS* mutant metastatic colorectal cancer patients

**DOI:** 10.18632/oncotarget.14810

**Published:** 2017-01-25

**Authors:** Mariantonietta Di Salvatore, Filippo Pietrantonio, Armando Orlandi, Marzia Del Re, Rosa Berenato, Ernesto Rossi, Marta Caporale, Donatella Guarino, Antonia Martinetti, Michele Basso, Roberta Mennitto, Concetta Santonocito, Alessia Mennitto, Giovanni Schinzari, Ilaria Bossi, Ettore Capoluongo, Romano Danesi, Filippo de Braud, Carlo Barone

**Affiliations:** ^1^ Unit of Clinical Oncology, Università Cattolica del Sacro Cuore, 00168 Rome, Italy; ^2^ Medical Oncology Department, Fondazione IRCCS Istituto Nazionale dei Tumori, 20133 Milan, Italy; ^3^ Clinical Pharmacology and Pharmacogenetic Unit, Department of Clinical and Experimental Medicine, University of Pisa, 56126 Pisa, Italy; ^4^ Laboratory of Clinical Molecular Biology, Institute of Biochemistry and Clinical Biochemistry, Università Cattolica del Sacro Cuore, 00168 Rome, Italy

**Keywords:** single nucleotid polymorphisms, bevacizumab, IL-8, eNOS, colorectal cancer

## Abstract

**Background:**

Predictive biomarkers of efficacy and toxicity of bevacizumab have not yet been validated. This study assessed the influence of IL-8, eNOS and VEGF-A polymorphisms in *RAS* mutated metastatic colorectal cancer patients receiving bevacizumab-based chemotherapy.

**Methods:**

120 patients treated with first-line combination FOLFOX6 plus bevacizumab were included. A historical cohort of 112 *RAS* mutated colorectal cancer patients treated with FOLFOX6 alone served as control group. The following SNPs were analyzed: IL-8 c.-251T>A; eNOS c.-786T>C and c.-894G>T; VEGF-A c.936C>T, c.958T>C, c.1154A>G and c.2578C>A. Correlation of SNPs, baseline IL-8 serum levels and bevacizumab-efficacy was done.

**Results:**

In the bevacizumab group, carriers of the IL-8 alleles c.-251TA+AA showed a shorter PFS (P=0.002) and OS (P=0.03) compared to TT alleles. Patients with pre-treatment IL-8 < 18.25 pg/ml showed significantly longer median PFS and OS (PFS: 10.9 vs 7.6 months, P=0.005; OS: 30.7 vs 18.2 months, P<0.001) compared to patients with IL-8 higher levels (>18,25 pg/ml). IL-8 c.-251TA+AA carriers had significantly higher IL-8 levels (P<0.0001). Multivariate analysis confirmed association of IL-8 polymorphism with PFS, and of IL-8 baseline levels with both PFS and OS. IL-8 SNP did not affect the outcome in the control group. The eNOS polymorphism c.-894G>T was found associated with higher severe toxicity (P=0.0002) in patients carrying the c.-894TT genotype.

**Conclusions:**

Although our data need prospective validation, IL-8 and eNOS SNPs may be have a role as predictive biomarkers for bevacizumab efficacy and toxicity.

## INTRODUCTION

To date, predictive biomarkers of efficacy and toxicity of bevacizumab have not yet been validated in metastatic colorectal cancer (mCRC) patients [1]. Some evidence suggests that clinical or radiological parameters might predict the efficacy of bevacizumab, but their clinical implementation has yet to be proven [2–4]. The use of predictive biomarkers might improve mCRC patients selection for bevacizumab-based treatment. This issue is particularly relevant for *RAS* mutated subset, due to limited availability of effective treatment options. Unfortunately, since angiogenesis is a multifactorial and host-mediated process, validation of such biomarkers is not always easy. The outcome of patients treated with bevacizumab-based therapies may be related to polymorphisms in genes involved in different aspects of angiogenesis, leading to changes in vascular endothelial growth factor (VEGF)-dependent and independent pathways. A role of VEGF single nucleotide polymorphisms (SNPs) has been suggested in colorectal cancer with controversial results [5, 6].

While VEGF-A is the target of bevacizumab, it is conceivable that resistance to treatment may also be linked to an angiogenic switch due to up-regulation of VEGF-independent pathways. For instance, interleukin-8 (IL-8) induces angiogenesis and increases endothelial permeability in absence of hypoxic environment [7–10]. The IL-8 c.-251T>A polymorphism seems to be associated to variations of promoter transcriptional activity and to higher levels of circulating IL-8 [11–15]. Moreover, VEGF up-regulate the expression of nitric oxide synthase [16, 17], resulting in the release of endothelium-derived nitric oxide and in the consequent stimulation of angiogenesis [18–21]. While the activation of VEGFR signaling pathway stimulates the eNOS leading to the production of the potent vasodilator nitric oxide, the inhibition of VEGF signaling might lead to decrease the nitric oxide concentrations, resulting in vasoconstriction. In fact, the eNOS polymorphisms were found to be associated to a higher risk of developing grade 3 hypertension in a group of patients treated with sunitinib [22]. However, in literature there are few and uncertain data regarding the eNOS role in bevacizumab-induced toxicity.

This study was aimed at exploring the role of SNPs in IL-8 (c.-251T>A), eNOS (c.-786T>C, c.-894G>T) and VEGF-A (c.936C>T, c.958C>T, c.1154A>G, c.2578C>A) as potential biomarkers of efficacy and toxicity of bevacizumab in *RAS* mutated mCRC. Moreover, it was evaluated the correlation of SNPs, IL-8 serum levels and bevacizumab efficacy.

## RESULTS

### Study population

One-hundred and twenty consecutive patients were enrolled from 2007 to 2010 in the bevacizumab group, and their clinical characteristics are reported in Table 1. Forty-seven (39.2%) patients experienced a partial response and 8 (6.6%) a complete response, while 53 (44.2%) had a stable disease and 12 (10%) had a PD. ORR was 45.8%, median PFS and median OS were 10 and 37.6 months, respectively. Among the main clinical characteristics, only the number of metastatic sites showed a statistically significant correlation both with PFS and OS in the univariate analysis. In particular, patients with >2 metastatic sites had a PFS and an OS significantly shorter (PFS: 6 vs 9.2 months, HR: 2.31, 95% CI 1.72-9.09, P=0.001; OS: 19.6 vs 29 months, HR: 2.77, 95% CI 2.27-12.5, P<0.001) compared to those with < 2 metastatic sites. Considering only grade 3-4 toxicities, 11 (9.1%) patients developed hypertension, 3 (2.5%) bleeding, 3 (2.5%) proteinuria, 3 (2.5%) venous thromboembolism, 1 (0.8%) arterial thromboembolism and 1 (0.8%) acute renal failure.

**Table 1:** Patients and disease characteristics in bevacizumab-treated group

Characteristics		N 120	%	Progression Free Survival			Overall Survival		
				HR	95% CI	P	HR	95% CI	P
**Age, years**	≤65	58	48.3						
	>65	62	51.7	1.30	0.87-2.29	0.59	1.27	0.67-2.75	0.55
**Sex**	Male	74	61.7						
	Female	46	38.3	0.91	0.79-1.26	0.70	1.02	0.82-1.40	0.79
**PS (ECOG)**	0-1	113	94.2						
	2	7	5.8	1.35	0.79-1.91	0.68	1.47	0.85-2.21	0.65
**Primary tumor site**	Right colon	68	56.6						
	Left colon	52	43.4	0.61	0.39-1.13	0.11	0.65	0.38-1.52	0.37
**Previous adjuvant treatment**	No	84	70.5						
	Yes	36	29.5	1.85	0.98-2.91	0.06	1.34	0.73-2.56	0.59
**N. of metastatic sites**	≤2	17	14.2						
	>2	103	85.8	2.31	1.72-9.09	***0.001***	2.77	2.27-12.5	***<0.001***
**Mucinous histotype**	No	101	84.2						
	Yes	19	15.8	1.71	0.85-3.38	0.12	2.23	0.91-3.31	0.10
**IL-8-251 T>A**	TT	43	36						
**rs4073**	TA	50	42						
	AA	27	22						
**TT+TA vs AA (recessive model)**				0.7	0.39-1.10	0.12	0.70	0.47-1.27	0.39
**TT vs TA+AA (dominant model)**				0.53	0.34-0.78	***0.002***	0.64	0.43-0.97	***0.03***
**eNOS c.-786T>C**	TT	33	27.5						
**rs2070744**	TC	60	50						
	CC	27	22.5						
**TT+TC vs CC (recessive model)**				1.08	0.68-1.70	0.70	0.98	0.60-1.50	0.96
**TT vs TC+CC (dominant model)**				1.3	0.85-2.20	0.2	0.92	0.59-1.43	0.73
**eNOS c.-894G>T**	GG	40	33						
**rs1799983**	GT	64	54						
	TT	16	13						
**GG+GT vs TT (recessive model)**				1.78	1.01-3.12	***0.049***	1.34	0.78-2.23	0.28
**GG vs GT+TT (dominant model)**				1.11	1.70-1.72	0.56	1.15	0.75-1.77	0.50
VEGF-A c.936C>T*	CC	43	61.4						
**rs3025039**	CT	25	35.7						
	TT	2	2.9						
**CC + CT vs TT (recessive model)**				1.04	0.25-4.35	0.955	1.39	0.23-7.96	0.74
**CC vs CT + TT (dominat model)**				1.38	0.35-5.38	0.642	1.60	0.27-8.47	0.64
VEGF-A c.958C>T*	TT	43	61.4						
**rs833061**	TC	14	20						
	CC	13	18.6						
**CC + CT vs TT (recessive model)**				1.54	0.73-3.27	0.25	1.32	0.43-4.56	0.57
**CC vs CT + TT (dominat model)**				1.03	0.51-2.12	0.92	1.21	0.41-3.40	0.72
VEGF-A c.1154A>G*	AA	32	45.7						
**rs1570360**	AG	28	40						
	GG	10	14.3						
**AA + AG vs GG (recessive model)**				1.83	0.86-3.45	0.07	1.81	0.85-3.12	0.11
**AA vs AG + GG (dominat model)**				1.23	0.75-1.32	0.45	1.32	0.65-1.36	0.56
VEGF-A c.2578C>A*	CC	23	32.8						
**rs699947**	CA	35	50						
	AA	12	17.2						
**CC + CA vs AA (recessive model)**				1.09	0.64-1.93	0.78	1.31	0.58-2.99	0.55
**CC vs CA + AA (dominant model)**				1.15	0.55-2.23	0.69	1.39	0.48-3.89	0.544

Association of clinical and genomic characteristics with PFS and OS in bevacizumab group.Bold: Clinical and SNPs characteristics. Italic and bold: statistical significant value.

*: Evaluated only in cohort 1 (Policlinico “Gemelli”).

Baseline characteristics of patients included in the control group are summarized in Table 2. Among the available clinical and pathological characteristics, only the number of metastatic sites showed a statistically significant correlation with OS in the univariate analysis (HR: 1.97, 95% CI 1.11-2.42, P=0.04). No significant correlation was found with PFS.

**Table 2:** Patients and disease characteristics in the control group (non-bevacizumab treated)

Characteristics		N 112	%	Progression Free Survival			Overall Survival		
				HR	95% CI	P	HR	95% CI	P
**Age, years**	≤65	50	44.6						
	>65	62	55.4	1.2	0.71-2.11	0.51	1.23	0.61-2.72	0.52
**Sex**	Male	58	51.7						
	Female	54	48.3	0.96	0.60-1.20	0.62	1.12	0.62-2.40	0.69
**PS (ECOG)**	0-1	102	91						
	2	10	9	1.15	0.66-2.10	0.66	1.77	0.86-3.21	0.43
**Primary tumor site**	Right colon	62	55.3						
	Left colon	50	44.7	0.69	0.39-1.23	0.18	0.65	0.29-1.61	0.37
**Previous adjuvant**	No	79	70.5						
	Yes	33	29.5	1.91	0.97-3.11	0.06	1.25	0.67-2.91	0.61
**N. of metastatic sites**	≤2	17	14.1						
	>2	95	95.9	1.45	0.89-2.34	0.41	1.97	1.11-2.42	***0.04***
**Mucinous histotype**	No	98	87.5						
	Yes	14	13.5	1.95	0.95-3.78	0.08	2.45	0.97-3.91	0.09
**IL-8-251 T>A**	TT	38	33.9						
**rs4073**	TA	45	40.1						
	AA	29	26						
**TT+TA vs AA (recessive model)**				0.91	0.82-1.15	0.71	0.88	0.79-1.23	0.69
**TT vs TA+AA (dominant model)**				0.95	0.87-1.11	0.66	0.87	0.84-1.12	0.64

Association of clinical and genomic characteristics with PFS and OS in control group.

Bold: clinical and SNPs characteristics. Italic and bold: statistical significant value.

### Correlation between genotypes and treatment outcomes

The genotyping analysis showed that all the SNPs were in Hardy-Weinberg Equilibrium and the relative frequencies of the selected and analysed SNPs are reported in Table 1 for bevacizumab group and in Table 2 for control group.

In the bevacizumab group, no statistically significant correlation was found between all analysed SNPs and ORR. Median PFS was significantly longer inpatients with IL-8 c.-251TT genotype as compared to those carrying the c.-251TA/AA genotypes (10 vs 8.2 months, HR: 0.53, 95% CI 0.34-0.78, P=0.002) (Figure 1A and Table 1); OS was also significantly longer inpatients with IL-8 c.-251TT *versus* c.-251TA/TT (33 vs 26.3 months, HR: 0.64, 95% CI 0.43-0.97, P=0.03) (Figure 1B and Table 1). Patients with eNOS c.-251TG/GG genotype showed a significant lower PFS as compared to those carrying the c.-894TT genotype (9 vs 10 months, HR: 1.78, 95% CI 1.01-3.12; P=0.049), but did not show a significantly worse OS (27 vs 37 months, HR: 1.34, 95% CI 0.78-2.23; P=0.2) (Table 1). All the other analyzed VEGF and eNOS SNPs failed to show any significant correlation with bevacizumab efficacy (Table 1).

**Multipanel Figure:**
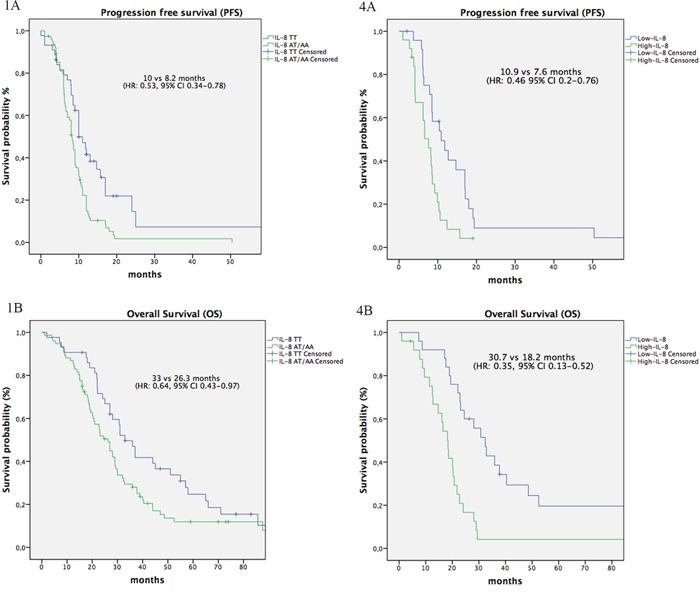
IL-8 SNPs and IL-8 serum levels influence PFS and OS Kaplan-Meier plots for PFS in patients with IL-8 TT genotype versus heterozygous TA and polymorphic homozygous AA genotypes **1A**. and in patients with IL-8-low serum level versus IL-8-high serum levels **4A**. Kaplan-Meier plots for OS in patients with IL-8 TT genotype versus heterozygous TA and polymorphic homozygous AA genotypes **1B**. and in patients with IL-8-low serum level versus IL-8-high serum levels **4B**.

The multivariate analysis was performed only with the significant clinical variable at the univariate analysis, i.e. number of metastatic sites. IL-8 c.-251AA/AT genotype and presence of >2 metastatic sites retained their significant association with worse PFS (P=0.0006, HR: 1.8, 95% CI 1.18-2.8; and P=0.015, HR: 1.8, 95% CI 1.12-2.9), while only the presence of >2 metastatic sites was independently associated with OS (P<0.001, HR: 2.4, 95% CI 1.44-.4.28). The eNOS c-894TT genotype was no longer associated with PFS.

**Figure 2:**
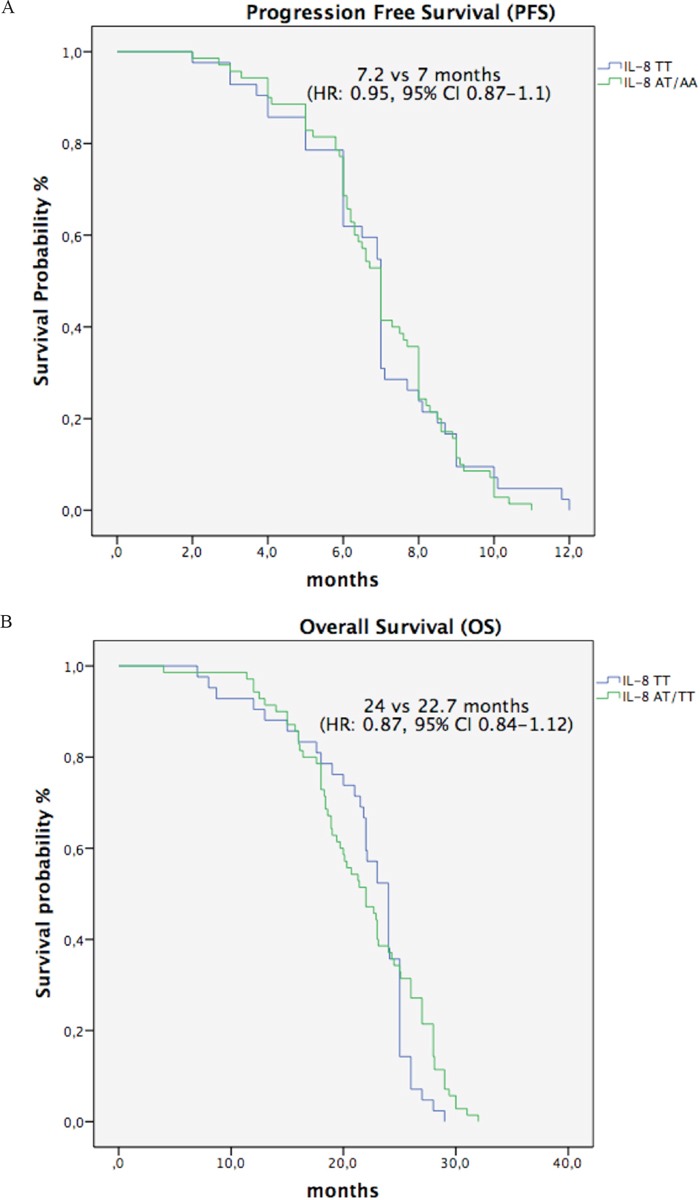
A-B. Kaplan-Meier plots for PFS (A) and OS (B) in control group patients with IL-8 TT genotype versus heterozygous TA and polymorphic homozygous AA genotypes

Given the independent prognostic value of IL-8 c.-251T>A, we assessed only this SNP in the historical control group of patients treated with FOLFOX only. No significant association with PFS or OS was detected (Figure 2A–2B), thus suggesting a potential predictive role of IL-8 SNP in patients treated we bevacizumab-based therapy.

Regarding toxicity, patients carrying the eNOS c.894TT genotype showed a statistically significant higher occurrence of hypertension and proteinuria when compared with GG + GT genotypes (P=0.0002) (Table 3). All analyzed VEGF SNPs, IL-8 c.-251T>A and eNOS c.-786T>C failed to show any significant correlation with toxicity (Table 3).

**Table 3:** Association of bevacizumab-related specific toxicity and candidate SNPs

SNPs	%	P
IL-8 -251 T>A		
TT+TA vs AA (recessive model)	14 *vs* 11.5	1
TT vs TA+AA (dominant model)	22 *vs* 10	0.06
eNOS -786 T>C		
TT+TC vs CC (recessive model)	12 *vs* 21	0.22
TT vs TC+CC (dominant model)	12 *vs* 15	0.77
eNOS -894 G>T		
GG+GT vs TT (recessive model)	8 *vs* 50	***0.0002***
GG vs GT+TT (dominant model)	5 *vs* 18	***0.04***
VEGF-A c.936C>T		
CC + CT vs TT (recessive model)	10 *vs* 11	0.93
CC vs CT + TT (dominat model)	9.5 *vs* 10	0.98
VEGF-A c.958C>T		
CC + CT vs TT (recessive model)	9 *vs* 12	0.73
CC vs CT + TT (dominat model)	8.5 *vs* 10	0.88
VEGF-A c.1154A>G		
AA + AG vs GG (recessive model)	8 *vs* 9	0.92
AA vs AG + GG (dominat model)	10.5 *vs* 10	0.98
VEGF-A c.2578C>A		
CC + CA vs AA (recessive model)	8 vs 8	1
CC vs CA + AA (dominat model)	9 vs 11	0.88

Relationship of polymorphisms with toxicity (hypertension and proteinuria).Italic and bold: statistical significant value.

### Functional significance of IL-8 c.251T>A for IL-8 serum levels

**Figure 3:**
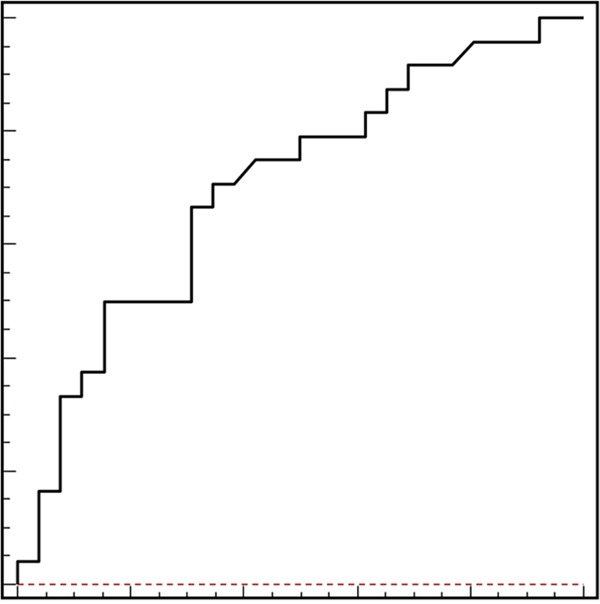
Receiver operating characteristics (ROC) curve analysis based on pre-treatment IL-8 serum levels In this model sensitivity was 80% (95% CI: 74.7–82.8) and specificity was 56% (95% CI: 51.9–79.1). Area under the curve was 0.71, P=0.004.

The median IL-8 serum level was 17 pg/mL (range 1.3-309.1 pg/mL). Using the ROC curve analysis, the baseline IL-8 serum level cut-off was 18.25 pg/mL (area under the curve 0.71, Figure 3), which was quite similar to the median value. For this reason, patients showing a pre-treatment IL-8 serum level >18.25 were classified as IL-8-high, and patients with pre-treatment level lower than 18.25 as IL-8-low. Median PFS and OS was significantly longer inIL-8-low patients as compared to IL-8-high patients (PFS: 10.9 vs 7.6 months, HR: 0.46, 95% CI 0.2-0.76, P=0.005; OS: 30.7 vs 18.2 months, HR: 0.35, 95% CI 0.13-0.52, P<0.001; Figure 4A and 4B, respectively). At the multivariate analysis including number of metastatic sites, only IL-8 levels >18.25 pg/mL had an independent association with worse PFS (P=0.0014, HR: 2.9, 95% CI 1.53-5.75) and OS (P=0.0002, HR: 3.2, 95% CI 1.7-5.9). Most importantly, serum levels of IL-8 in patients with IL-8 c.-251 TT genotype (median level 4.7 pg/mL) was statistically lower as compared to those with TA (median level 29.3 pg/mL) or AA genotypes (median level 32.2 pg/ml) (H: 17.0, DF: 1 P<0.0001). Interestingly, in a parallel cohort of 50 healthy volunteers enrolled in the two Institutions as controls, the median IL-8 level was 4 pg/mL (range 1.5-35.7 pg/mL) and presence of IL-8 SNP was not correlated with variations of serum levels (H: 3.4, DF: 1, P=0.67).

## DISCUSSION

Several data on angiogenesis-related SNPs as predictive biomarkers of bevacizumab-based treatment were obtained from retrospective series and mostly involved VEGF [23]. For instance, VEGF c.-1498 TT SNP was associated with shorter PFS in mCRC patients treated with first-line FOLFIRI and bevacizumab [5]. However, a subsequent prospective validation study failed to confirm this hypothesis [6]. In line with these data, our study failed to show any significant impact of VEGF-A c.936C>T, c.958T>C, c.2578C>A and c.1154A>G. This study was aimed at showing whether SNPs related to VEGF-independent pathways might influence the efficacy of bevacizumab-based treatment in patients with *RAS* mutated mCRC. We focused on *RAS* mutated tumors since recent retrospective analyses of randomized trials seem to suggest a relatively smaller benefit from antiangiogenic agents in this patients’ subgroup with limited treatment options [24, 25]. Thus, the discovery of predictive biomarkers of efficacy of antiangiogenic treatments in this patient population is an unmet clinical need. It is well known that IL-8 can induce an angiogenic switch [7–10], allowing the “escape” from VEGF-targeted treatment [26]. We showed for the first time that the IL-8 polymorphism c.-251 “A” allele (dominant model) was associated with significantly shorter PFS and OS. The multivariate model confirmed an independent correlation with PFS (P=0.01), but not with OS. This apparent discrepancy is not surprising, since PFS is widely accepted as a surrogate endpoint in first-line treatment, due to dilution of effects on OS by post-progression treatments. Similar observations on this IL-8 SNP were recently shown in patients with breast cancer treated with bevacizumab-based chemotherapy [27, 28] and in exudative macular degeneration after bevacizumab treatment [29]. Since the “A” allele of the IL-8 SNP is associated with increased IL-8 production [11], a plausible biological explanation of our results is that patients carrying the A allele might benefit less from bevacizumab because of higher IL-8 serum levels. In support of the role of IL-8 as factor of resistance to bevacizumab in metastatic colorectal cancer patients, a better clinical outcome was reported when IL-8 baseline levels were lower [30, 31]. On the contrary, Kopetz *et al*. showed that high IL-8 levels were associated with a shorter PFS and increased tumor volume [32]. In our study, we confirm that higher IL-8 serum levels were associated with shorter PFS and OS. Moreover, we demonstrate for the first time the functional effect of IL-8 c.-251T>A SNP in cancer patients. In fact, we showed a statistically significant correlation between the polymorphic “A” allele and higher IL-8 serum levels, achieving the proof-of-concept that IL-8 might play a key role in the intrinsic resistance to bevacizumab in mCRC. Since the presence of polymorphic “A” allele did not influence IL-8 serum levels in healthy volunteers, we hypothesize that the pro-inflammatory state caused by the metastatic process may enhance IL-8 transcription in genetically predisposed patients. The genetic variation may also drive treatment-acquired resistance due to IL-8-driven pro-inflammatory switch. In this scenario, IL-8 may represent not only a prognostic biomarker in patients treated with anti-angiogenic therapy, but also a therapeutic target.

Regarding the eNOS gene, preclinical studies showed that eNOS c.-894G>T variant has a functional effect on eNOS protein, leading to a reduced NO production [17]. Recently, Ulivi et al. found that eNOS c.-894GT polymorphism may also predict efficacy of bevacizumab based-therapy in mCRC [33]. In our study, the multivariate analysis seemed to exclude a potential impact of eNOS on efficacy outcomes. However, we also focused on toxicity and showed that eNOS c.-894TT genotype was associated with significantly higher rate of grade 3-4 hypertension and proteinuria as compared to others. It must be pointed out that potential bevacizumab-specific toxicities can be serious and contraindicate the use of this agent in patients at risk. Not surprisingly, alteration in the eNOS pathway may be correlated with the most frequent toxicities of bevacizumab, such as hypertension and proteinuria [34, 35]. In this context, in patients carrying eNOS c.-894 polymorphic “T” allele, lower basal levels of eNOS may increase the risk of hypertension induced by VEGF blockade [36–38].

Despite the biological consistency of our results, our study is limited by small sample size and retrospective evaluation. Therefore, it should be considered only as exploratory study. However, the absence of prognostic impact of IL-8 SNPs in the control group, allow to formulate the hypothesis that this factor may be a predictor of efficacy to bevacizumab-based treatment. However, the study of polymorphisms in alternative pathways of angiogenesis affords an original cue for future research on biomarkers predictive for bevacizumab activity and toxicity and may open the way to anti-IL-8 strategies to overcome primary or acquired resistance to bevacizumab in selected patients.

## MATERIALS AND METHODS

### Patients selection

This study was conducted at two Italian Institutions: the cohort 1 comprised 70 patients treated at Policlinico “A. Gemelli” of Rome; the cohort 2 comprised 50 patients treated at Fondazione IRCSS Istituto Nazionale dei Tumori of Milan. Both cohorts included only *RAS* mutated patients treated with first-line combination of FOLFOX6 plus bevacizumab (bevacizumab group). The study was approved by the local Ethics Committee and written informed consent was required before study procedures. Main inclusion criteria were: histologically confirmed diagnosis of mCRC, age ≥ 18 years, presence of at least one measurable lesion according to RECIST 1.0, no prior treatments for metastatic disease, ECOG performance status between 0 and 2, and acceptable bone marrow, liver and renal functions. We excluded patients with serious concomitant illness that could affect the treatment outcome or survival. Treatment was continued until disease progression, occurrence of unacceptable toxicity or consent withdrawal. A historical cohort of 112 *RAS* mutated patients treated at Policlinico “A. Gemelli”, from January 2002 until November 2007, with first-line FOLFOX alone served as control group.

### Single nucleotide polymorphisms genotyping

The following SNPs were analysed: IL-8 c.-251T>A; eNOS c.-786T>C and c.-894G>T; VEGF-A c.936C>T, c.958T>C, c.1154A>G and c.2578C>A. Given their promising results, IL-8 and eNOS SNPs were analyzed in the whole series. VEGF SNPs were explored exclusively in cohort 1 and, given the non significant results in this training set, further analyses on the whole series was abandoned. Blood samples were stored at -20°C and used for molecular analyses. DNA wasi extracted with Maxwell (R) 16 System from Promega (Madison, USA), using a specific kit (AS1010-Maxwell 16 Blood DNA purification kit) that allows the simultaneous extraction of DNA from 16 blood samples. Samples were analysed using the polymerase chain reaction-restriction fragment length polymorphism (PCR-RFLP) technique. Once extracted, DNA from each sample was amplified by PCR with primers specific for each SNP. PCR products were digested by restriction enzymes (FastDigest ® Restriction Enzymes, Fermentas, USA) specific for each polymorphism. The fragments obtained from each cut were then separated on the basis of molecular weight by electrophoresis on 3% agarose gel in 1x TAE buffer (40 mM tris [pH 7.6], 20 mM acetic acid and 1 mM EDTA), colored with ethidium bromide and visualized with ultraviolet light. The genotyping analysis was performed by laboratory personnel and patient clinical outcomes were blinded to laboratorists.

### IL-8 serum levels quantification

Pre-treatment serum samples were available only for patients in cohort 2. IL-8 levels were quantified by ELISA, with the use of a Quantikine ELISA Human CXCL8/IL-8 Immunoassay kit (R&D Systems a biotechne brand, Minneapolis, MN), according to the manufacturer’s protocol.

### Statistical analysis

All polymorphisms were examined for deviation from Hardy-Weinberg equilibrium (HWE) by comparing actual allelic distributions with those expected from HWE using a χ2-test. No formal statistical hypothesis testing neither adjustment for multiple comparison were performed because this study was exploratory in nature and aimed to generate hypothesis for future studies. Therefore, only descriptive statistics were derived. Progression free survival (PFS) was defined as the time from treatment start to progressive disease (PD) or death. Overall survival (OS) was defined as the time from treatment start to death or last follow up. Overall response rate (ORR) was evaluated by RECIST version 1.0 criteria and tumor assessments were repeated every 12 weeks until PD. Bevacizumab-related toxicities were assessed at each cycle according to National Cancer Institute Common Toxicity Criteria (version 3.0). The association between each gene polymorphism with response and toxicity was evaluated by Fisher’s exact test. The Kaplan-Meier method and log-rank test were used to estimate the role of each variable in predicting the hazard ratio (HR) for disease progression and death. Clinical variables including: age (≤65 vs>65), histology (mucinous versus non-mucinous), primary site, ECOG performance status (0-1 vs 2), number of metastatic sites (≤2 vs >2) and previous adjuvant treatment. The correlation of clinical characteristics and SNP status with survival was assessed in univariate analyses according to the dominant and recessive genetic models. Cox proportional hazard model was adopted in the multivariate analysis, including as covariates variables significantly correlated with survival in the univariate analyses (P<0.05). Receiver operating characteristics (ROC) curve analysis was performed to determine a cutoff value for pre-treatment IL-8 levels using median PFS as endpoint. The Kruskal-Wallis test was used to correlate IL-8 serum levels with SNP -251 T/A. All analyses were two-sided, and statistical significance was defined by a P value of <0.05. Analyses were performed using SPSS for MAC (version 22.0.0).

## Abbreviations

IL-8: Interleukine 8
mCRC: metastatic colorectal cancer
ORR: Overall response rate
OS: Overall Survival
PD: Progression disease
PFS: Progression free survival
SNPs: single nucleotide polymorphisms
VEGF: vascular endothelial growth factor.
